# Metastatic Breast Cancer with BRCA Mutation Discovered By Next-Generation Sequencing Responding to Olaparib

**DOI:** 10.7759/cureus.1337

**Published:** 2017-06-11

**Authors:** Wajeeha Rizvi, Phu Truong, Quoc Truong

**Affiliations:** 1 Internal Medicine, University of Kansas School of Medicine - Wichita; 2 Cancer Center of Kansas, University of Kansas School of Medicine - Wichita

**Keywords:** invasive ductal breast cancer, next-generation sequencing, metastatic breast cancer, brca, olaparib

## Abstract

Breast cancer susceptibility genes 1 and 2 (BRCA1 and BRCA2) mutations are associated with hereditary breast and ovarian cancer syndromes (HBOC). However, certain individuals with breast cancer do not meet high-risk factors for hereditary breast cancer screening based on age, family history, and biology of malignancy.

We present a patient with relapsed breast cancer who developed progressive disease with significant tumor burden causing a recurrent pleural effusion. Next-generation sequencing (NGS) done on a tumor biopsy was positive for the BRCA2 mutation. Olaparib was initiated with a resolution of the pleural effusion and a significant decrease in the size of the malignant lymphadenopathy and pulmonary lesions.

There are numerous reports of comprehensive molecular profiling improving access to therapy, most notably for lung cancer, as well as melanoma. However, this has not been widely utilized for breast cancer. However, in our case, NGS provided our patient with an effective therapy and should be considered for the future management of metastatic breast cancer.

## Introduction

According to the American Cancer Society, about one in eight women in the United States will develop invasive breast cancer over the course of her lifetime. In 2017, an estimated 252,710 new cases of invasive breast cancer are expected to be diagnosed [1]. A minority of these breast cancers are associated with hereditary breast and ovarian cancer syndromes (HBOC). Even though BRCA1 and BRCA2 are well known for their cancer risk, specific cancer risk by BRCA1 or BRCA2 germline missense variants is not well established [2]. These germline missense variants are also known as variants of unknown significance (VUS), some missense mutations have been classified as deleterious or likely deleterious. Molecular genetic testing is used to identify germline variants to diagnose BRCA1 and BRCA2 HBOC. Treatment of BRCA 1-2 positive cancers is similar to that of other cancers. However, prophylactic bilateral mastectomy, bilateral oophorectomy, and chemoprevention are recommended by the National Comprehensive Cancer Network [3].

Among novel therapies, Olaparib is an orally active poly (ADP-ribose) polymerase (PARP) inhibitor that was approved in 2014 by U.S. Food and Drug Administration (FDA) for the treatment of BRCA-positive advanced ovarian cancer [4]. It was recently investigated in the OlympiAD Phase III trial and has shown significant improvement in progression-free-survival (PFS) compared to chemotherapy in BRCA mutated metastatic breast cancer [5].

In this article, a case of BRCA positive metastatic breast cancer determined through next generation sequencing (NGS) is presented to highlight consideration of comprehensive molecular profiling to help clinicians treat metastatic breast cancer. 

## Case presentation

This is a 55-year-old post-menopausal female who was diagnosed with a Stage IIIC (T3N2aM0) invasive ductal carcinoma of the right breast with multiple positive right axillary lymph nodes in 2014. The malignancy was positive for estrogen receptor (ER) 95%, progesterone receptor (PR) 70%, human epidermal growth factor receptor 2 (HER2) 1+, and the marker of proliferation antigen KI-67 (MKI67) 25%. Metastatic workup was negative for distant metastases. The patient chose to have bilateral mastectomies and therapy was initiated with standard cytotoxic chemotherapy consisting of Adriamycin/Cytoxan followed by weekly Taxol in 2015. Post-mastectomy radiation was completed since the original breast cancer was greater than 5 cm. Hereditary breast and ovarian cancer genetic analysis was considered but was denied by insurance due to lack of high-risk criteria.

The patient was maintained on an oral aromatase inhibitor (AI). She tolerated the AI well for one year, but in 2016, she developed increased shortness of breath and right supraclavicular swelling. Chest computed tomography (CT) was done revealing massive lymphadenopathy and a right pleural effusion, causing superior vena cava syndrome (SVC). She received radiation therapy as well as placement of a stent, which improved the SVC syndrome.

**Figure 1:**
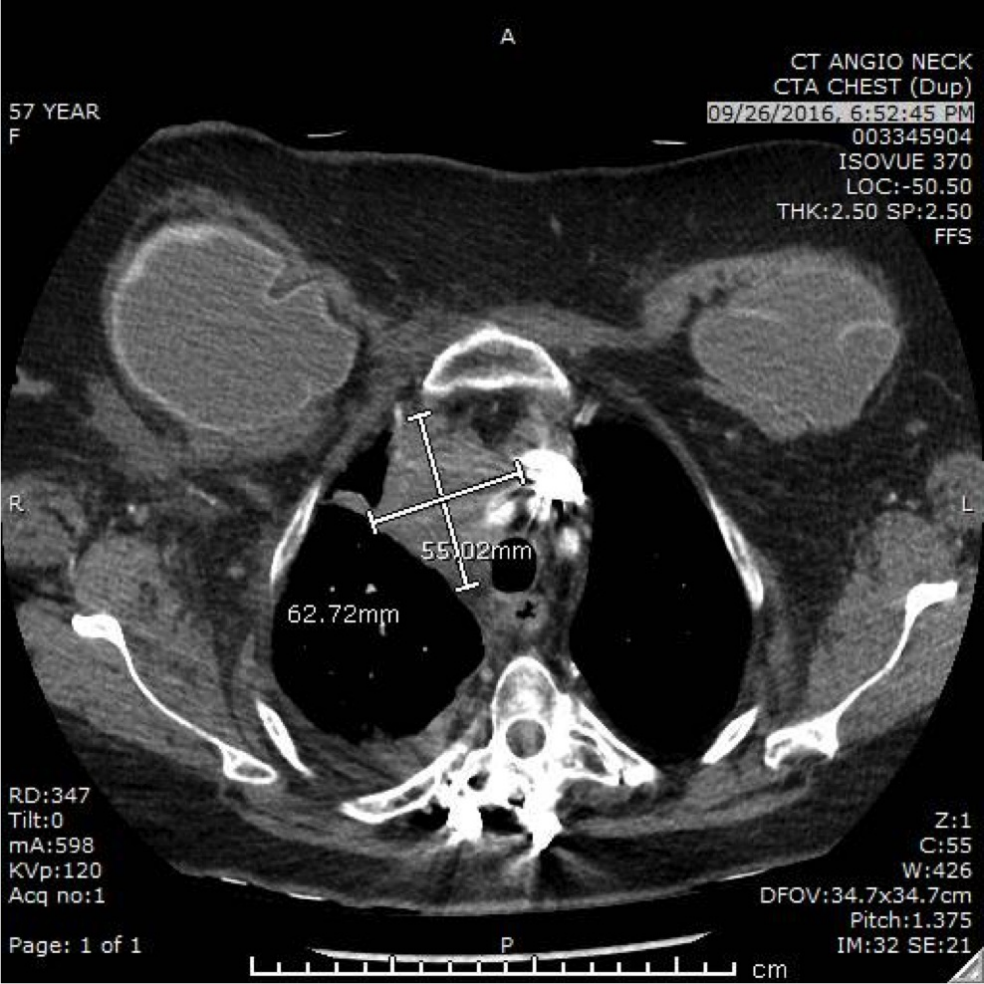
Computed tomography angiography (CTA) scan of the chest before initiation of Olaparib therapy

A combination of fulvestrant and palbociclib was initiated; however, after two months, there was evidence of disease progression on CT angiography of the chest (Figure 1). Therapy was changed to a single agent, nab-paclitaxel, and the patient clinically improved with a mild reduction of pulmonary lesions after two cycles of therapy. However, after the administration of the seventh cycle, there was worsening disease progression with the advancement of pleural effusion, lymphadenopathy, and new bone and liver metastases.

**Figure 2:**
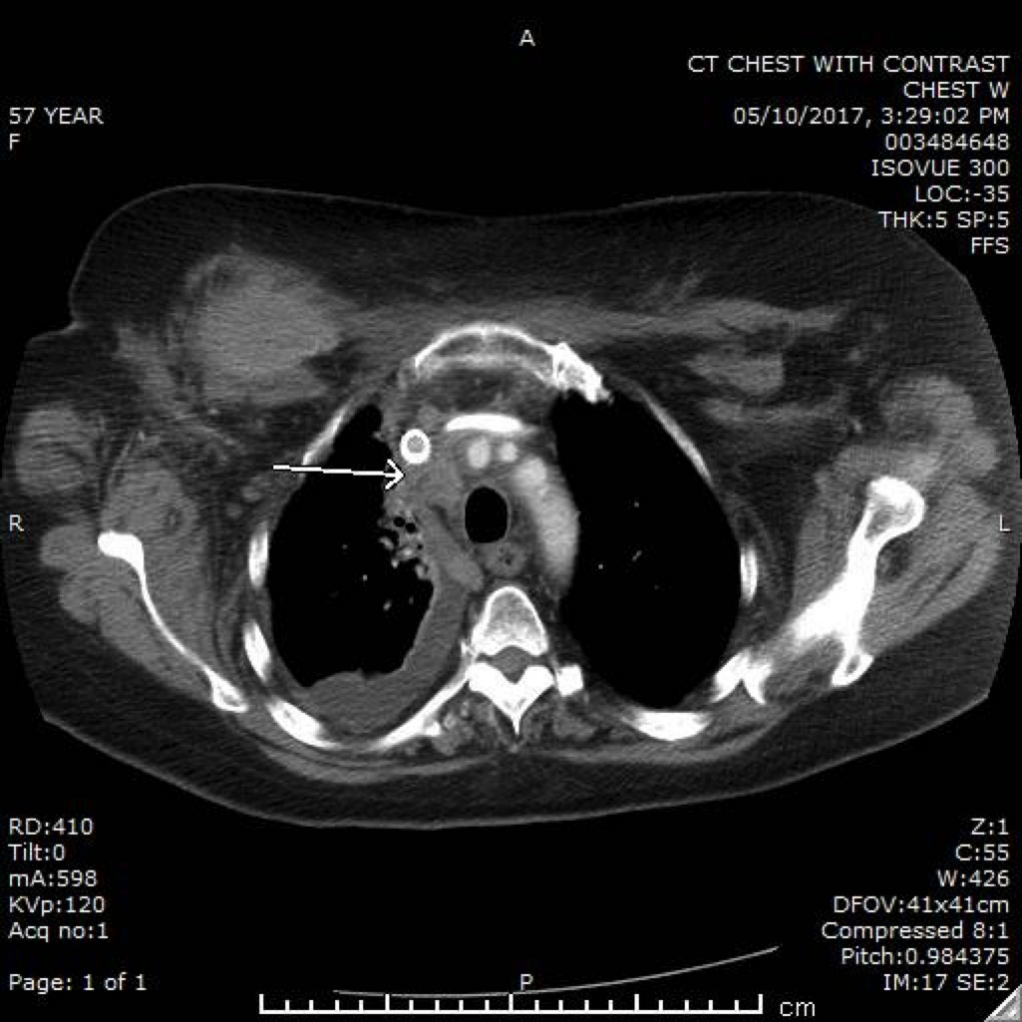
CT chest after six months of therapy on Olaparib showing improvement in the pleural effusion and lymphadenopathy

Biopsy of the liver was consistent with recurrent breast cancer with the same hormonal/HER2 profile. NGS was performed on biopsy specimen through Foundation 1 (F1). The F1 panel was positive for BRCA2 heterozygosity and ataxia-telangiectasia mutated (ATM) gene. With the mutations in mind, the patient was started on oral targeted therapy with Olaparib. After a few months, repeat CT imaging was done which revealed resolution of the pleural effusion and improvement of the lymphadenopathy, as well as the bone and liver metastases (Figure 2). Currently, she is tolerating chemotherapy well, except for mild fatigue.

## Discussion

BRCA1 and BRCA2 genes pose significant risk factors in developing metastatic breast cancer. Other genes, like ATM, checkpoint kinase 2 (CHEK2), BRCA1 interacting protein C-terminal helicase 1 (BRIP1), and partner and localizer of BRCA2 (PALB2) are also associated with increased risk for breast cancer [6]. Several studies are being conducted to improve targeted therapy and ensure better patient outcomes and PFS. However, insurance and Medicare reimbursements for testing are currently only approved for certain high-risk individuals. Another option for these patients is to utilize molecular profiling to identify certain mutations that oral targeted therapies are currently available. One of these novel therapies, Olaparib, which is approved for BRCA-mutated ovarian cancer, has recently demonstrated statistically significant and clinical improvement in metastatic breast cancer compared to physician’s choice of chemotherapy. The Phase III OlympiAD trial focused on poly (ADP-ribose) polymerase inhibitors (Olaparib) therapy on metastatic breast cancer patients who had HER2 negative disease with BRCA1 or BRCA2 mutations and revealed remarkable progress in disease control and PFS. 

## Conclusions

BRCA1 and 2 are widely studied genetic risk factors for breast cancer. Diagnostic and therapeutic advancement is the key to improving the five-year survival and treatment outcomes. For individuals with metastatic breast cancer who progress on standard therapies, one should consider comprehensive molecular profiling. Our innovative approach using F1 panel testing for recurrent breast cancer revealed BRCA2 and ATM mutations, hence, leading to a successful response to oral chemotherapy with Olaparib based on Phase 3 OlympiAD trial. Maximum efforts are needed to ascertain cancer cell susceptibilities, which can help in the treatment of this malignancy that afflicts many women in the U.S. 
